# Central Control of Feeding Behavior by the Secretin, PACAP, and Glucagon Family of Peptides

**DOI:** 10.3389/fendo.2017.00018

**Published:** 2017-02-07

**Authors:** Revathi Sekar, Lei Wang, Billy Kwok Chong Chow

**Affiliations:** ^1^School of Biological Sciences, The University of Hong Kong, Hong Kong, China

**Keywords:** secretin, PACAP, and glucagon family peptides, hypothalamus, feeding behavior, energy homeostasis, metabolic diseases

## Abstract

Constituting a group of structurally related brain-gut peptides, secretin (SCT), pituitary adenylate cyclase-activating peptide (PACAP), and glucagon (GCG) family of peptide hormones exert their functions *via* interactions with the class B1 G protein-coupled receptors. In recent years, the roles of these peptides in neuroendocrine control of feeding behavior have been a specific area of research focus for development of potential therapeutic drug targets to combat obesity and metabolic disorders. As a result, some members in the family and their analogs have already been utilized as therapeutic agents in clinical application. This review aims to provide an overview of the current understanding on the important role of SCT, PACAP, and GCG family of peptides in central control of feeding behavior.

## Introduction

Secretin (SCT), pituitary adenylate cyclase-activating peptide (PACAP), and glucagon (GCG) family or SCT family, a group of short peptides that were classified based on their structural homology and named after the first hormone discovered, include SCT, PACAP, vasoactive intestinal peptide (VIP), GCG, glucagon-like peptide-1 (GLP-1), GCG-like peptide-2 (GLP-2), glucose-dependent insulinotropic polypeptide (GIP), growth hormone–releasing hormone (GHRH), and peptide histidine isoleucine (PHI) or peptide histidine methionine (1). While sharing some biological functions, each of these hormones possesses distinct physiologic actions such as the involvement of SCT in water homeostasis (2). Similar to their ligands, receptors mediating functions of the peptides are structurally related and are grouped in the class B G protein-coupled receptors (3, 4). Recent pharmacological interests for these receptors have paved way for novel therapeutic strategies for intervention of pathological conditions (5–8). For instance, liraglutide and exenatide (GLP-1 receptor agonists) are being used for treating diabetes (5).

Feeding is a complex behavior involving the integration of homeostatic systems that sense energy balance with hedonic (reward) behavior (9). Areas in the central nervous system (CNS) that appear to be important for regulation of feeding behavior are distributed across regions of forebrain and caudal brain stem (10, 11). Of particular significance is the hypothalamic neural circuitry, long known to be involved in the control of energy homeostasis in response to various endocrine, nutritional, metabolic, and thermal signals. The role of hypothalamus in energy homeostasis regulation has been discussed in detail in several review articles (10, 12–16). Arcuate nucleus (ARC), paraventricular nucleus (PVN), ventromedial hypothalamus (VMH), dorsomedial hypothalamus (DMH), and lateral hypothalamus (LH) are hypothalamic regions with reciprocal connections that are known to be involved in regulation of food intake and energy homeostasis. In the ARC above the median eminence, neurons expressing neuropeptide Y (NPY) and agouti gene-related protein (AgRP) stimulate food intake and are found medially, while pro-opiomelanocortin (POMC) (precursor of α-melanocyte-stimulating hormone; α-MSH) and cocaine- and amphetamine-regulated transcript (CART) induce anorexia and are coexpressed in the neuronal population of lateral ARC. These neurons project to the PVN which controls feeding and provides preganglionic autonomic output to the brainstem. NPY reduces energy expenditure and stimulates food intake through the Y1 and Y5 receptors in the PVN. Conversely, CART inhibits NPY-induced feeding (17–19). Also, POMC and its products such as α-MSH suppress feeding behavior. AgRP is an endogenous antagonist of α-MSH and, therefore, increases food intake and weight. Other hypothalamic peptides, such as melanin-concentrating hormone and hypocretin, are orexigenic in nature and are expressed in distinct populations of neurons in the lateral hypothalamic area (20–22). Another important site in regulation of feeding behavior is caudal brain stem. In the brain stem, neurons in the nucleus tractus solitarius (NTS) and dorsal motor nucleus of the vagus (DMV) receive and integrate inputs from vagus nerve, which is involved in sensing nutrient accumulation in the stomach and the duodenum (23). Highly interconnected with the hypothalamus, brainstem regulates responses to fasting through ascending projections to the hypothalamus (10) and short-term satiety signals through descending projections from the hypothalamus (24). Apart from the brain circuits regulating hunger and satiety, nuclei embedded within the mesolimbic reward circuitry [ventral tegmental area (VTA) and nucleus accumbens (NAc)], which are well known for their importance in reinforcing properties of drugs of abuse and natural rewards (25), as well as nuclei in the amygdale and hippocampus are involved in the rewarding effects of food (26).

The SCT family peptides and their receptors have been found to exist in various brain regions, including hypothalamus, implying their neuroactive functions (27, 28). Most of them have also been found to be important in the central regulation of energy balance (29). Therefore, in the sections below, we have reviewed in detail the role of SCT family hormones in the central control of feeding behavior.

## Secretin

Secreted from the S-cells of the duodenum in response to acid, SCT is a 27-amino acid peptide and the first ever hormone discovered (30). SCT primarily functions in the gastrointestinal tract to stimulate bicarbonate secretion from the pancreas to neutralize acid (31). A century-long research has gone into studying the gastrointestinal functions of SCT, and only recently it has been found as a neuropeptide (2, 32–36). As we summarized before (28), SCT was found to be expressed in multiple brain sites, including PVN, supraoptic nucleus (SON), and ARC of hypothalamus, NTS of brainstem, central amygdala (CeA), hippocampus, and cerebral cortex (37, 38). SCT receptor (SCTR) expressions were also found in PVN, SON, and ARC of the hypothalamus, among other brain regions (39). In spite of contradictory evidences in the past (40–42), our laboratory has recently confirmed an anorectic effect of peripheral and central SCT in mice (39), supported by our findings that intracerebroventricular (i.c.v.) and intraperitoneal (i.p.) injections of SCT reduced food intake in wild-type mice. This observation was absent in SCTR knockout (SCTR^−/−^) mice, further confirming the specificity of SCT–SCTR axis in regulating feeding behavior.

The previous review from our lab (28) has concluded that circulating SCT might not cross the blood–brain barrier (BBB) for specifically exerting its anorectic effect (38, 39), although SCT has been shown to be able to cross the BBB (43). Thus peripheral SCT, through SCTR in the intestinal vagal afferents, communicated to the brain to bring about satiety control without causing conditioned taste aversion (CTA) (39, 44). However, a very recent study has provided another piece of the puzzle on the appetite regulation by peripheral SCT. By using male oxytocin (OXT)-monomeric red fluorescent protein 1 (mRFP1) transgenic rats, the study showed that i.p. SCT (100 µg/kg), apart from inducing a reduction in food intake, stimulated mRFP1 fluorescence (OXT indicator) in the dorsal division of the parvocellular PVN (dpPVN) and increased mRFP1-positive granules in the axon terminals of dpPVN OXT neurons in the NTS. An upregulation of c-Fos expression was also observed in the NTS and OXT neurons of dpPVN. Therefore, peripheral SCT might regulate feeding behavior, at least in part, *via* an OXTergic pathway from the dpPVN to the NTS (45).

Consistently, central SCT is also able to reduce food intake. It has been shown that central SCT induced c-Fos immunoreactivities in the ARC and PVN of hypothalamus. In the ARC, POMC neurons were found to be colocalized with activated c-Fos as well as with SCTR. An increase in POMC and a reduction in AgRP transcript levels were observed in the ARC after i.c.v. and i.p. SCT (39, 46). In addition, it has been reported that central and peripheral SCT increased melanocortin-4 receptor (MC4R) mRNA in the PVN, and administration of MC4R antagonist, SHU9119, in the PVN reduced the anorectic effect of central and peripheral SCT, indicating the involvement of melanocortin system (39, 46). We previously showed that K^+^-induced depolarization of hypothalamic explants released SCT endogenously through voltage-gated sodium and calcium channels, suggesting that this endogenous SCT could function as a neurotransmitter in the region (34, 35). Taken all together, SCT has been suggested as a pleiotropic regulator of feeding behavior and neuroendocrine signaling in the hypothalamus. Microinjection of SCT into the CeA has been recently shown to reduce cumulative food intake through cAMP-activated protein kinase pathway, while electrophysiological recordings indicated that SCT may exert its anorectic actions, at least in part, by modulation of spontaneous firing of CeA neurons (47).

Absence of a truly functional SCTR antagonist is partly a hindrance for studying the region-specific (hypothalamus or amygdala) role of SCT in modulating food intake behavior. Recent establishment of several animal models such as SCT^−/−^ or SCTR^−/−^as well as the SCT^fl/fl^ for cell-specific knockout of SCT should help to reveal the relationship between SCT and anorectic functions. Research on food intake modulations by SCT has been initiated for the past few years but still requires further efforts to test its potential in therapeutic interventions.

## Pituitary Adenylate Cyclase-Activating Peptide

Pituitary adenylate cyclase-activating peptide, a 38-residue peptide hormone, was first isolated from ovine hypothalamic tissue as a hypophysiotropic peptide that would activate adenylate cyclase in cultured rat pituitary cells (48) and is widely distributed in the body including brain, gonads, and adrenal gland (49, 50). Various regions of the brain like central thalamic nuclei, amygdaloid complex, and hippocampus expressed PACAP and yet the most abundant expression was found in the ARC, PVN, VMH, and SON of the hypothalamus (50, 51), which has been well documented to be involved in appetite regulation. Double labeling studies have shown PACAP to be colocalized with POMC neurons of the ARC but not POMC neurons within the brain stem (52). Receptors for PACAP (PAC1R and/or VPAC2R) were also found to be expressed in approximately half of POMC neurons (53, 54) and a significant proportion of NPY neurons (53, 54). Consistent with its distribution pattern in the brain, i.c.v. administration of PACAP was found to reduce food intake by several studies (55–57). Notably, in rat, Mizuno’s group showed that PACAP induced a long-lasting reduction of food consumption (58). As we have reviewed before (28), a study showed that 100 nM PACAP increased POMC mRNA, α-MSH tissue content as well as α-MSH release in mediobasal hypothalamic explants (53, 54). Another study further revealed that central PACAP stimulated c-Fos expression in POMC neurons and increased POMC and MC4R transcripts in the ARC; PACAP-specific receptor (PAC1R) knockout mice had lower POMC transcript levels in the ARC compared to wild-type animals and pre-injection of SHU9119, MC3R/MC4R antagonist, abolished the hypophagic effects of i.c.v.-PACAP, suggesting that PACAP might act through PAC1R receptor and then melanocortin system to exert its anorectic effect (57). Using AtT20PL, a clone of the AtT20 mouse corticotroph tumor cells stably transfected with rat POMC 5′ promoter-luciferase fusion gene, PACAP and VIP were found to increase the POMC promoter activity and expression *via* a PKA-independent intracellular signaling pathway (59).

Intra-PVN injection of PACAP has been shown to reduce food intake as well as lead to significant reductions in meal size, duration, and total time spent eating (60), consistent with the loss-of-function study showing that there was a pronounced hyperphagia in PVN-lesioned animals (61), and PACAP receptor-specific study showing that the feeding behavior was primarily controlled by PAC1R and this receptor subtype was abundantly distributed in the PVN (60). Although PVN has been suggested as the predominant site of action for PACAP-mediated hypophagia, the PACAP stimulation of the VMH may serve primarily to stimulate energy expenditure (60). Direct acute injection of PACAP into the VMH, a region heavily expressing PAC1R, was able to inhibit food intake for 6 h which could be reversed by the PAC1R antagonist. Intra-VMH injection of PACAP also increased POMC mRNA in the ARC while not affecting NPY and AgRP mRNA levels (62). Previous studies have shown that PACAP is capable of modulating the activation of ionotropic glutamate receptors (63, 64). Recently, it has been shown that glutamatergic signaling *via* NMDA receptors was required for the hypophagic effects of intra-VMH PACAP (65). A novel model of binge behavior that could temporally separate homeostatic feeding from palatable food-driven (hedonic) feeding behavior (66), has shown that the microinjection of PACAP into NAc mimics the actions of GABA agonists and reduces the intake of palatable food without altering homeostatic feeding, while microinjection of PACAP into the VMH mimics the actions of AMPA by decreasing homeostatic feeding without altering hedonic feeding. Furthermore, it has been shown that transcript levels of PACAP in the VMH was regulated according to energy status, as fasting reduced and high-fat diet increased PACAP expression in VMH (67).

Recently, monosynaptic interactions of PACAP-expressing neurons in the VMH with appetite-suppressing POMC neurons of the ARC have been shown (68). Nevertheless, this study also showed, using channel rhodopsin-assisted circuit mapping, that appetite-stimulating AgRP neurons in the ARC had excitatory PACAPergic afferents originating within the PVN. Incidentally, a previous study has reported that PACAP could stimulate NPY neurons to elevate cytosolic Ca^2+^ levels (69). These exciting new findings suggested that, based on their neuroanatomical location, PACAP could stimulate both orexigenic and anorexigenic effects (70). A very recent study showed a role of PACAP/PAC1 signaling during light-regulated feeding behavior (71), adding more complexity on the appetite regulation by PACAP in the hypothalamus. Therefore, further detailed studies are required to understand how these pathways interact under various energy states and converge to modulate feeding behavior.

Apart from its anorectic role, local PVN as well as i.c.v. infusion of PACAP-38 significantly induced plasma glucose concentration, endogenous glucose production, and c-Fos immunoreactivity in the autonomic neurons in the PVN. These neurons project to preganglionic sympathetic neurons in the spinal cord and are involved in hepatic glucose production (72). Furthermore, PACAP has been shown to interact with other peptides to modulate feeding behavior. Central administration of PACAP provokes increases in hypophysiotropic neurohormones in the hypothalamus, such as vasopressin, GnRH, somatostatin, and CRF (73, 74). In chicks (75) and goldfish (76), it has been shown that anorectic effect of central PACAP was inhibited by CRH receptor antagonist, astressin, and α-helical CRH_(9–41)_, respectively, and GnRH2 has been found to mediate CRH-signaling pathway in goldfish (77). PACAP mRNA was found to be colocalized with steroidogenic factor-1in the VMN and leptin signaling was required for normal PACAP expression in these cells, while blocking of endogenous central PACAP signaling attenuated leptin-stimulated hypophagia and hypothermia (67). Consistently, i.p. administration of PACAP has been found to suppress appetite with a decrease in plasma ghrelin and an increase in plasma GLP-1 and leptin (78, 79). For its integrative role in glucose and energy homeostasis, PACAP receptor subtype-specific agonists and/or antagonists are being considered as potential therapeutic agents for metabolic disorders in addition to appetite disorders.

## Vasoactive Intestinal Peptide

Distributed throughout the gastrointestinal tract (80, 81) and CNS including cerebral cortex, suprachiasmatic nucleus (SCN), and PVN of the hypothalamus and thalamus (82, 83), VIP is a 28-amino acid peptide hormone known for its role in vasodilation and hypotension acting through VPAC1R and VPAC2R receptors. VPAC1R expression has been found mainly in the cerebral cortex and hippocampus, while VPAC2R was expressed in the thalamus, midbrain and in the PVN and SON magnocellular cells and SCN of the hypothalamus (83, 84). Previous studies have demonstrated that plasma VIP concentrations increased following either a carbohydrate meal or water loading (85), and short-term fasting altered the VIP levels in the hypothalamus and other brain regions, suggesting a potential for VIP in modulating appetite and food intake (86). Indeed, i.c.v. administration of VIP induced anorexia in chicken (87, 88), goldfish (89, 90), and rat (91). Disruption in food intake and metabolic rhythm occurred in VIP and VPAC2R knockout mice (92). VIP could possibly stimulate hypothalamic–pituitary–adrenal (HPA) axis as intra-PVN injection of VIP increased secretion of ACTH and corticosterone (93), possibly by activating the CRH neurons (94). Additionally, stimulation of hypothalamic explants by VIP significantly stimulated the release of α-MSH (91), suggesting VIP could also work through the activation of melanocortin system to inhibit food intake. As mentioned earlier, cell line studies with transfected rat POMC promoter-luciferase fusion gene have shown that VIP induces POMC promoter activity through a PKA-independent pathway (59). Recently, VIP knockout mice were shown to have a disrupted pattern of circadian feeding behavior resulting in a significantly reduced nocturnal/diurnal feeding along with reduced body weight and fat mass accumulation (78, 79). The study also showed that, in VIP knockout mice, the release of anorexigenic hormones, such as GLP-1, leptin, PYY, and insulin was altered in both fasting and post-prandial conditions, revealing a possibility of VIP cross-talking with other hormones to inhibit food intake. However simultaneously, orexigenic hormones were also found to be altered suggesting the role of VIP in both anorexigenic and orexigenic effects (78, 79). There were also reports indicating an absence of anorectic effects by VIP as i.c.v. injection of VIP did not influence appetite in fasted mice (57) and administration of VIP receptor antagonist in the PVN had no effect on food intake in rats (94). With such contrasting evidences, the role of VIP on appetite control remains currently unclear.

## Glucagon

Derived from proglucagon that contains sequences for GLP-1 and GLP-2, GCG is a 29-amino acid peptide hormone secreted by pancreatic α cells in response to low blood glucose. Counteracting hypoglycemia, it antagonizes insulin action by stimulating hepatic glucose synthesis and mobilization. In contrast to its peripheral action, recent reports have shown that hypothalamic action of GCG inhibited hepatic glucose production (95, 96). Its role in energy homeostasis and metabolism has been reviewed in detail before (97–99). Also as we have reviewed before (28), peripheral GCG induced satiety in humans (100, 101) and in rats (102, 103) without causing CTA (104); GCG affected meal size rather than meal interval (105); and hepatic vagal afferents were found to mediate this effect of peripheral GCG (106). In addition, another study reported that subcutaneous injection of GCG was able to reduce appetite and through immunohistochemical analysis c-Fos expression could be detected in the NTS, area postrema (AP), and CeA but absent in the ARC, PVN, and DMH regions of the hypothalamus, suggesting the involvement of brainstem and amygdala in the appetite control by peripheral GCG (107). GCG receptor (GCGR) distribution has been found in the dorsal vagal complex (DVC) of brainstem (108). A recent study investigating the role of GCG in high-protein feeding has demonstrated that elevated circulating GCG found during high-protein feeding acted in the DVC through GCGR-dependent PKA–Erk1/2–K_ATP_ signaling cascade to contribute to the effect of high-protein feeding (109). However, relatively low levels of GCGR were also found in the hypothalamus, and it is noteworthy that circulatory GCG has been shown to suppress glucose sensing *via* LH, DMH and VMH neurons of hypothalamus, hence the possibility of hypothalamic activation in appetite control by peripheral GCG could not be excluded (110). While the mechanisms behind the anorectic effect of peripheral GCG were not fully understood, there were also reports suggesting that peripheral GCG increased food intake (111).

Central administration of GCG has been observed to induce anorexia in rats (112, 113), chicks (114, 115), and sheep (116) with much higher anorectic effect appeared after i.c.v. GCG than its peripheral effect in rats (112). Although the mechanisms underlying satiety regulation by central GCG remains a mystery, a previous review has discussed several possibilities. For example, it suggested the stimulation of hypothalamic corticotropin-releasing factor and activation of HPA axis to be involved in the anorectic effect of GCG (117). Microinjection of GCG into the LH reduced appetite and stimulated sympathetic activity (118). Although high levels of immunoreactive GCG and relatively low levels of GCGR were found in the hypothalamus (108), the role of hypothalamic neurons in appetite control by GCG was not clear, until a recent report proved that central GCG reduced appetite through hypothalamic pathway (119). Hypothalamic GCG activated GCGR to stimulate downstream PKA pathway in the hypothalamic ARC, as i.c.v. co-infusion of the GCGR antagonist des-His^1^-[Glu^9^] GCG amide or the PKA inhibitor H-89 negated the ability of central GCG to induce anorexia. And as the downstream factor of PKA, central GCG injection also reduced protein levels of Ca^2+^-calmodulin-dependent protein kinase kinase β (CaMKKβ) and its downstream target phosphorylated AMP-activated protein kinase (AMPK) in the ARC. AMPK was upstream Acetyl-CoA carboxylase (ACC), and it was hence observed that the injection of a constitutively active AMPK virus in the ARC was able to recover the decrease of ACC caused by central GCG and attenuated the anorectic effects of GCG. Consistent with above findings as well as the co-localization of the GCGR in AgRP neurons of the ARC (95), a significant reduction in the expression of AgRP was observed after central GCG injection. Diet-induced obesity abolished the anorectic effects of GCG but it was restored by molecular inhibition of CaMKKβ in the ARC *via* adenoviruses encoding dominant negative CaMKKβ. Central GCG, therefore, exerted its acute anorectic effects through PKA/AMPK/CaMKKβ-dependent pathways in the ARC and CaMKKβ mediated its obesity-induced hypothalamic resistance (119). Taken together, even though several aspects of the central GCG’s role in controlling feeding behavior have been discussed above and also by other studies, further research is still required to get better understanding and shed light on the beneficial effects by coordination of central GCG with other hormones such as insulin and GLP-1.

## Glucagon-Like Peptide-1

Processed from the preproglucagon (PPG), GLP-1 is a 30-amino acid peptide hormone secreted from the L-cells of the intestinal epithelium in response to nutrient intake (120) and acts as an incretin along with GIP stimulating glucose-dependent insulinotropic action primarily (121, 122). As the most explored hormone for its role in feeding behavior among the SCT family of peptides, a plethora of research has brought clarity and understanding on the peripheral and central effects of endogenous and exogenous GLP-1 on food intake and glycemic control as reviewed in several reports (123–126). Acting with GLP-1 receptors (GLP-1Rs), GLP-1 altered food intake behavior through various neural substrates, including hypothalamus (ARC, PVN, and LH), hindbrain nuclei [parabrachial nucleus (PBN), area postrema (AP), medial NTS (mNTS)], ventral hippocampus (vHP), and nuclei embedded within the mesolimbic reward circuitry (VTA and NAc). Within these areas, the diverse neural circuitry involved in feeding control by GLP-1 has been recently reviewed in detail (127). Following nutrient intake and entry into the gastrointestinal tract, peripheral GLP-1, endogenously released from the L-cells, acted in a paracrine manner on the GLP-1R that was expressed on dendritic terminals of the celiac and gastric branches of the vagal afferents which innervated the intestine to exert its satiation effect. This vagal activation *via* vagal-to-NTS glutamatergic signaling relayed signals to nodose ganglion for activating the NTS neurons in the brain (128). On GLP-1 stimulation, GLP-1R on the intestinal vagal afferents also stimulated pancreatic insulin secretion *via* vago-vagal reflex (128, 129). Indeed, it was possible that peripheral GLP-1 through a vagal afferent-independent pathway crossed the BBB to directly activate the central GLP-1R of NTS. But a recent finding that after subdiaphragmatic vagal deafferentation, i.p. injection of GLP-1 did not affect the size of the first meal suggested otherwise (130). Although there was a question about whether peripheral GLP-1 was able to act directly in the brain, in the case of peripheral injections of long-acting GLP-1 analogs like liraglutide and exendin-4 (Ex-4), it was quite clear that they brought about reductions in food intake, at least partly by crossing the BBB and directly acting on the brain regions (131–133).

Central GLP-1 was a potent modulator of blood glucose utilization along with an anorectic effect (134). GLP-1 was found to be produced endogenously in the caudal nucleus of NTS and in the ventrolateral medulla while GLP-1R expression was found in the PVN, ARC, and DMH of hypothalamus as well as in the NTS, AP, and PBN of brainstem (135) revealing the functional sites of GLP-1. In the hypothalamus, i.c.v.-GLP-1 induced c-Fos expression in the PVN (134), while intra-PVN GLP-1 produced satiety (136, 137). PVN GLP-1R activation caused anorexia (138) and selective blockade of PVN GLP-1R resulted in hyperphagia and weight gain (139). GLP-1 (and/or GLP-1 analogs) primarily activated CRH and nesfatin-1 neurons (and to a lesser extent OXT neurons) in the PVN (139). Additionally, HPA axis and catecholamine release was stimulated by central GLP-1 and GLP-1R in the PVN (140). Although it has been reported that central GLP-1 induced an acute dose-dependent anorectic effect and this effect was abolished after the damage of ARC (141), there were inconsistent findings supporting and negating the anorectic actions of GLP-1 injected into the ARC (142). Thus, it warrants further research to clarify the discrepancy. Intra-LHGLP-1 induced hypophagia that was short latency and short lasting, while liraglutide reduced food intake for 24 h after its injection into LH (143). Local injections of GLP-1 into the VMH or the DMH also resulted in short latency, short-lasting (1–2 h) hypophagia while there was no effect for liraglutide in these areas (144). In spite of multiple action sites of GLP-1 in the hypothalamus, which indicated its indispensable role in regulating appetite *via* neuroendocrine pathways, some negative side effects of centrally administrated GLP-1 could not be easily ignored. For example, i.c.v. GLP-1 induced CTA effect (145) and central GLP-1R participated in LiCl-induced CTA effect as GLP-1R antagonist abolished the response. Bilateral lesions of CeA reversed the aversive behavior but not anorectic effect while bilateral lesions in the PVN was *vice versa* indicating that PVN was important for inhibition of food intake and CeA for aversive effect of GLP-1 (137, 146). However, it is still in need of more research to increase the knowledge of regional-specific effects of GLP-1 and expand the positive effect on clinical applications.

Caudal brain stem processing has been found to be sufficient for carrying out the various effects of peripheral and hindbrain GLP-1R activation (147). While all three nuclei of the DVC of the hindbrain (NTS, AP, DMV) expressed the GLP-1R, it was NTS GLP-1R expressing cells that were physiologically and pharmacologically more significant in modulating food intake behavior (127). It was unclear if NTS-PPG neurons expressed GLP-1R, while there was a report showing that they did not respond to GLP-1R ligands (148). Hindbrain GLP-1R activation has been found to suppress food intake *via* PKA-mediated suppression of AMPK activity and simultaneous activation of p44/42 MAPK in NTS neurons (149). In the same report, Ex-4, a GLP-1R agonist, activation of the same signaling cascades in GT1-7 neuronal cells and in NTS lysates supports the view that these pathways occur in GLP-1R-expressing neurons. As the mechanisms underlying the anorectic function of NTS GLP-1R activation are being studied, there were complimentary reports suggesting that intra-mNTSGLP-1 analogs mediated nausea responses and hence the anorexic effect was induced at least in part by reducing motivation to feed or by eliciting pica response and illness like behavior (150). GLP-1 producing neurons in the NTS projected to lateral and medial PBN in rodents (151, 152) and consistently, GLP-1Rs have been found to be expressed in the lateral PBN (lPBN) (153). Activation of GLP-1R by microinjection of Ex-4, in lPBN resulted in reduced food intake and body weight along with reduced ingestion of palatable food and motivation to work for it (152, 154). Taken all together, these studies have provided another aspect of appetite control by GLP-1through brainstem pathways.

Glucagon-like peptide-1 neurons in the hindbrain projected directly to the NAc and VTA of the mesolimbic reward system (155), and both these areas expressed GLP-1R (153). GLP-1R activation in these nuclei led to reduction in reward-motivated behaviors for palatable food (156), alcohol (157), and cocaine (158). Injection of Ex-4 into the VTA or into the NAc core or shell reduced body weight, intake of regular chow, and intake of high-fat diet while not inducing pica response or CTA (155, 156, 159). Furthermore, injection of GLP-1R antagonist into mesolimbic nuclei increased the intake of regular chow, high-fat diet, liquid sucrose meal, and alcohol suggesting a role of endogenous GLP-1 in these nuclei (152, 157, 159). Interestingly, blockade of GLP-1Rs in the NAc-core increased high-fat diet intake, whereas blockade of shell GLP-1Rs did not show the effect (152), indicating a difference in actions of endogenous GLP-1 in the core and shell of NAc. Furthermore, it has been recently found that GLP-1R activation in NAc core suppresses food intake by increasing glutamatergic AMPA/Kainate signaling (160). *Ex vivo* electrophysiological studies revealed that GLP-1R activation in NAc core activates GABAergic medium spiny neurons predominantly by a presynaptic, AMPA/kainate-mediated glutamatergic mechanism and does not involve dopamine signaling. Consistently, *in vivo* intra-NAc core GLP-1R activation-induced food intake suppression and body weight reduction were attenuated by blockade of AMPA/Kainate receptors but not NMDA receptors (160).

Recently, central GLP-1 has been found to increase dopamine signaling in amygdala (161). Central activation of GLP-1R increased tyrosine hydroxylase, rate limiting enzyme for dopamine synthesis in the VTA (161, 162), which might contribute to increase in somatodendritic release of dopamine in the VTA (163). VTA dopaminergic neurons also projected to the amygdala where central Ex-4 acutely upregulated dopamine turnover and amygdale-dopamine receptor activation-induced satiety (161). Hence, novel VTA-amygdala dopamine circuit is being proposed as one of the underlying circuits involved in the anorectic effect of GLP-1 (161). In addition, a recent study has revealed that GLP-1R activation in vHP robustly reduced feeding, high-fat palatable food in particular while antagonizing GLP-1R in vHP region increased feeding (164), suggesting vHP as another target of GLP-1 controlling appetite. Surprisingly, the knockdown of GLP-1R did not alter the food intake in mice (165) and consistently body weight gain induced by high-fat feeding was also unaltered in GLP-1R knockout mice (166). These indicated that there were other compensatory factors that could make a good combination with GLP-1. Indeed for instance, GLP-1 has been identified to be one of the downstream mediators of leptin and leptin has been suggested to enhance the central GLP-1 activity (167).

The use of Ex-4 and liraglutide, two GLP-1R agonists, for treatment of diabetes produced small yet significant reductions in body weight (168–171). GLP-1 also induced weight loss after bariatric surgery (172). Dual agonism of GLP-1R/GCGR (173) and very recently triagonism with GLP-1R/GIPR/GCGR (174) have been found to have potent effects to reverse obesity in rodents. A GLP-1R agonist has also been found to have therapeutic effects on alcohol use disorders in mice (175). With these increasing therapeutic advantages, the hunt for GLP-1R agonists and combinatorial integrative effect of GLP-1 with other hormones is increasing to obtain maximum efficiency in therapeutic treatments.

## Growth Hormone–Releasing Hormone

Named after its stimulating effect of pituitary growth hormone, GHRH or growth hormone-releasing factor is a 44-amino acid hypothalamic peptide (176–179). Predominantly in the hypothalamic region, GHRH containing cell bodies are located primarily in the ARC along with the DMH and VMH (180, 181). It has been shown that i.p. injection of GHRH failed to alter food intake in rodents (182) while peripheral injection of chicken GHRH inhibited feeding behavior in chicks (183), suggesting the complexity of the peripheral GHRH in feeding regulation. The i.c.v. injection of GHRH stimulated food intake at doses that did not stimulate GH release (182, 184) while the i.c.v. injection of GHRH suppressed feeding behavior in rats at a higher dose (185), suggesting that not only peripheral and central GHRH could have different effects on appetite control but different doses of GHRH might also cause different feeding behavior.

As we reviewed before (28), the cell bodies of ARC-GHRH neurons had varied projection sites with their nerve terminals being at perifornical region, lateral preoptic area and SCN/medialpreoptic area (SCN/MPOA) (186) and mapping studies have identified SCN/MPOA as the central site of action for GHRH to induce orexigenic effects (184, 187). Orexigenic effect of GHRH has been found to be photoperiod sensitive which was consistent with the role of SCN/MPOA in controlling circadian rhythms (188). The i.c.v.-GHRH stimulated a dose-dependent increase and suppression of feeding during the light and dark phases of photocycle, respectively, in rats (189), and intra-SCN/MPOA-injected GHRH antiserum reduced dark onset feeding (190) with selective suppression of protein intake (191). Collectively, these reports suggested a role of endogenous GHRH in the regulation of circadian feeding rhythm. GHRH action was found to stimulate protein intake with no effects on carbohydrate intake (192). The observation that intra-ARC injected morphine stimulated protein intake was reversed by intra-SCN/MPOA pretreatments with GHRH antiserum (193), further confirming the significant role of GHRH in the regulation of protein intake. Additionally, Intra-PVN injection of opiate antagonist inhibited the effect of i.c.v.- (194) and SCN/MPOA-injected GHRH (192) suggesting a role of opiates in GHRH-induced feeding behavior. Transcriptional profiling of hypothalamic glucose-sensing neurons along with electrophysiological studies has revealed that hypoglycemia activated GHRH neurons (195). The electrical patterns that controlled the hypothalamic GHRH neurons have remained elusive while recently somatostatin has been found to bring about irregular suppression of the neuronal activity of GHRH neurons (196). In summary, it is the important role in GH secretion along with its involvement in nutritional and circadian feeding behavior that has made GHRH vital in integration and coordination of diverse aspects related to metabolism, growth and nutrient regulation (190, 197).

## Other Peptides

Other peptides from SCT family also shared a certain degree of functional similarities on the feeding behavior. Derived from the PPG, GLP-2 was known to have anorectic effect (198, 199). Central but not peripheral injection of GLP-2 reduced food intake and stimulated c-Fos expression in the ARC, PVN, DMH, VMH, and LH (200). The anorectic effect by central GLP-2R activation was reversed in MC4R knockout mice suggesting the involvement of melanocortin system and specific deletion of GLP-2R in the ARC POMC neurons resulted in increased intake (199).

Unlike its incretin counterpart GLP-1, GIP alleviated obesity through increased energy expenditure but did not affect food intake (201–203). However, recent evidences have shown the modulation of hypothalamic gene expression by i.c.v.-GIP (204). The i.c.v. administration and intra-PVN or -CeA injection of PHI decreased food consumption in overnight-deprived rats (205). These peptides’ roles in appetite regulation has not been well understood, hence further research is warranted for clarity in understanding the role of these peptides in neuromodulation of feeding behavior.

## Conclusion

This review has focused on central roles of the SCT, PACAP and GCG family of peptides in regulating food intake behavior. With members of the family such as GLP-1 exhibiting significant therapeutic advantage, SCT family of peptides have been considered as an important group of hormones in neural appetite modulation. With the exception of GHRH which is an orexigenic peptide, members of the SCT family of peptides mostly exhibit anorectic roles with GLP-1 and PACAP being most-studied among all the peptides. Central sites of action, effects on specific aspect of feeding behavior control, and the central circuitry recruited to carry out these effects vary widely among the SCT family of peptides and they have been summarized in Table 1. More information on the site-specific endogenous actions of these peptides on food intake modulation and the neural pathways involved along with the mechanistic insights is still unclear. Of the neural circuitry involved, melanocortin system (206), stimulated mainly through activation of POMC neurons, is the most common neural pathway through which these peptides, at least partially, exert their anorectic effect. Yet, the combinatorial effect of these peptides, along with the mechanisms and the neural pathways involved in such a scenario, is important to understand if they have synergistic additive effects on food intake reduction. Importance of this research is clearly evident as a novel monomeric peptide triagonist acting on GLP-1, GIP, and GCG receptors (174) has been found to be the most effective among existing pharmacological agonists/strategies in reversing obesity in mice (207, 208).

**Table 1:** **Secretin (SCT), pituitary adenylate cyclase-activating peptide (PACAP), and glucagon (GCG) family of peptides in neural regulation of appetite**.

Peptide	Primary physiological function	Effect on feeding behavior	Central site of action	Central mode of action
SCT	Stimulates bicarbonate release from pancreas and water homeostasis regulation	Anorectic	Hypothalamus: ARC and PVN Brainstem: NTS Amygdala: CeA	Activates oxytoxinergic pathway from the dpPVN to the NTS Stimulates POMC neurons in ARC Activates melanocortin system in PVN Modulates spontaneous firing of CeA neurons

PACAP	Strong modulator of hypothalamic magnocellular neurons	Anorectic	Hypothalamus: ARC, PVN, NAc and ventromedial hypothalamus (VMH)	Stimulates POMC neurons of ARC and activates melanocortin system through MC4R in PVN Increases ARC-POMC expression by intra-VMH administration and acts through glutamatergic signaling *via* NMDA receptors in VMH Acts through excitatory PACAPergic afferents originating within the PVN to AgRP/NPY neurons of ARC activating NPY neurons (possibly orexigenic effect) In NAc, mimics actions of GABA and reduces hedonic but not homeostatic feeding In VMH, mimics AMPA and reduces homeostatic but not hedonic feeding

Vasoactive intestinal peptide	Vasodilation and hypotesion	Not clear	Hypothalamus: PVN and SCN	Stimulates HPA axis and melanocortin system

GCG	Induces glucose release	Anorectic	Hypothalamus: ARC, LH Brainstem: DVC	Acts through GCGR-dependent PKA–Erk1/2–K_ATP_signaling cascade in DVC Stimulates HPA axis Acts through PKA/AMPK/CaMKKβ-dependent pathways and reduces AgRP in ARC

GCG-like peptide-1 (GLP-1)	Incretin	Anorectic	Hypothalamus: PVN, ARC, LH, DMH, and VMH Brainstem: mainly NTS, lPBN as well as AP, DMV Mesolimbic reward system: VTA and NAc Amygdala: CeA Hippocampus: vHP	Activates NTS via vagal to NTS glutamatergic signaling Stimulates HPA axis in PVN Causes CTA in CeA Reduces motivation to feed or elicits pica response in NTS Acts through NTS to lPBN circuit to activate lPBN leading to reduced ingestion of palatable food and motivation to work for it in lPBN Reduces reward-motivated intake of palatable food, alcohol and cocaine in VTA and NAc (no pica response or CTA) Acts in NAc core neurons through GLP-1R activation *via* presynaptic, AMPA/kainate-mediated glutamatergic mechanism Acts through VTA to amygdala dopamine circuit Reduces feeding and high-fat palatable food in vHP

GCG-like peptide-2 (GLP-2)	Intestinal function	Anorectic	Hypothalamus: ARC	Stimulates POMC and activates melanocortin pathway

Growth hormone–releasing hormone	Growth hormone release	Orexigenic	Hypothalamus: ARC, DMH, SCN/MPOA	Participates SCN/MPOA mediated circadian feeding stimulation Stimulates protein intake Induces opiate-involved feeding behavior

Peptide histidine isoleucine	Prolactin regulation	Anorectic	Hypothalamus: PVN Amygdala: CeA	Acts through oxytocin/vasopressin system in PVN and activates PVN OT neurons by intra-CeA infusion

*ARC, arcuate nucleus; PVN, paraventricular nucleus; NTS, nucleus tractus solitarius; CeA, central amygdala; POMC, pro-opiomelanocortin; dpPVN, dorsal division of the parvocellular PVN; VMH, ventromedial hypothalamus; MC4R, melanocortin-4 receptor; AgRP, agouti gene-related protein; NPY, neuropeptide Y; SCN, suprachiasmatic nucleus; HPA axis, hypothalamic–pituitary–adrenal axis; LH, lateral hypothalamus; DVC, dorsal vagal complex; AMPK, AMP-activated protein kinase; CaMKKβ, Ca^2+^-calmodulin-dependent protein kinase kinase β; DMH, dorsomedial hypothalamus; lPBN, lateral parabrachial nucleus; AP, area postrema; DMV, dorsal motor nucleus of the vagus; VTA, ventral tegmental area; NAc, nucleus accumbens; vHP, ventral hippocampus; CTA, conditioned taste aversion; MPOA, medialpreoptic area*.

Recent research on the neurobiology of food intake behavior has taken a leap with the advances in neuro-technology for mapping, manipulating, and monitoring molecularly defined cell types that are exponentially expanding our understanding into appetite modulating neural circuits (209). Although there is a huge gap between our current knowledge of the SCT family peptides and valuable clinical implications of these peptides for disease treatment, significance on the clinical applications of these peptides has already been evident by the significant therapeutic advantage by GLP-1R agonists in human (210, 211). In overweight or obese individuals, GLP-1R agonists have been shown to produce clinically relevant reductions in weight, body mass index, and waist circumference (212, 213). Liraglutide 3.0 mg day^−1^ has been approved for weight management in the US on December 23, 2014 (214), and in the EU on March 23, 2015 (215). Novel combinatorial hormone therapies are being researched at the pre-clinical stage for obesity treatment (216), wherein the novel unimolecular GLP-1R/GIPR/GCGR triagonist at a very low dose of 2 nmol/kg decreases cumulative food intake and reduces the body weight of HFD-mice by 18.3% and is more effective than any other available therapy (174). While the application prospect of SCT family peptides is exciting, some aversive consequences brought by injection of these peptides should not be easily ignored. Further regional and functional specific research as well as the understanding on combinational effects of SCT family peptides needs to be greatly enhanced. Future studies with the new tools that give neurobiologists opportunity to rigorously examine neural circuits in modulating feeding behavior should provide insights and novel therapeutic approaches to combat pathophysiological conditions related to appetite disorders and obesity.

## Author Contributions

RS contributed to manuscript preparation and manuscript definition of intellectual content. LW also contributed to manuscript preparation and followed by manuscript editing and revision. These two authors contributed equally to this work and should be listed as co-authors. BC, as the corresponding author, approved the final version of the manuscript.

## Conflict of Interest Statement

The authors declare that the research was conducted in the absence of any commercial or financial relationships that could be construed as a potential conflict of interest.
