# Corrigendum: Central Control of Feeding Behavior by the Secretin, PACAP, and Glucagon Family of Peptides

**DOI:** 10.3389/fendo.2018.00395

**Published:** 2018-07-18

**Authors:** Revathi Sekar, Lei Wang, Billy Kwok Chong Chow

**Affiliations:** School of Biological Sciences, The University of Hong Kong, Hong Kong, Hong Kong

**Keywords:** secretin, PACAP, and glucagon family peptides, hypothalamus, feeding behavior, energy homeostasis, metabolic diseases

In the published article, there was an error regarding the contributions. Revathi Sekar and Lei Wang should be listed as equal contributors.

The authors apologize for this error and state that this does not change the scientific conclusions of the article in any way.

## Conflict of interest statement

The authors declare that the research was conducted in the absence of any commercial or financial relationships that could be construed as a potential conflict of interest.

